# *DNMT3B7* Expression Promotes Tumor Progression to a More Aggressive Phenotype in Breast Cancer Cells

**DOI:** 10.1371/journal.pone.0117310

**Published:** 2015-01-21

**Authors:** Patrick R. Brambert, Daniel J. Kelpsch, Rabia Hameed, Charmi V. Desai, Gianfranco Calafiore, Lucy A. Godley, Stacey L. Raimondi

**Affiliations:** 1 Department of Biology, Elmhurst College, Elmhurst, Illinois, United States of America; 2 Section of Hematology/Oncology, Department of Medicine, The University of Chicago, Chicago, Illinois, United States of America; University of North Carolina School of Medicine, UNITED STATES

## Abstract

Epigenetic changes, such as DNA methylation, have been shown to promote breast cancer progression. However, the mechanism by which cancer cells acquire and maintain abnormal DNA methylation is not well understood. We have previously identified an aberrant splice form of a DNA methyltransferase, *DNMT3B7*, expressed in virtually all cancer cell lines but at very low levels in normal cells. Furthermore, aggressive MDA-MB-231 breast cancer cells have been shown to express increased levels of *DNMT3B7* compared to poorly invasive MCF-7 cells, indicating that DNMT3B7 may have a role in promoting a more invasive phenotype. Using data gathered from The Cancer Genome Atlas, we show that *DNMT3B7* expression is increased in breast cancer patient tissues compared to normal tissue. To determine the mechanism by which *DNMT3B7* was functioning in breast cancer cells, two poorly invasive breast cancer cell lines, MCF-7 and T-47D, were stably transfected with a *DNMT3B7* expression construct. Expression of *DNMT3B7* led to hypermethylation and down-regulation of E-cadherin, altered localization of β-catenin, as well as increased adhesion turnover, cell proliferation, and anchorage-independent growth. The novel results presented in this study suggest a role for DNMT3B7 in the progression of breast cancer to a more aggressive state and the potential for future development of novel therapeutics.

## Introduction

Breast cancer is the most common form of cancer among women in the United States, excluding non-melanoma skin cancers[1]. In 2013, more than 230,000 women in the United States were diagnosed with invasive breast cancer and an additional 60,000 were diagnosed with *in situ* disease. Approximately 40,000 women died of breast cancer last year, second only to lung cancer. Five-year survival rates vary significantly depending on the level to which a tumor has progressed at the time of diagnosis. Today, cases of localized breast cancer have a 99% survival rate. However, that rate falls to 24% when the cancer has metastasized to distant organs[1]. These differences in survival rates highlight the importance of determining the mechanisms responsible for tumor progression to a more aggressive and metastatic phenotype.

Cancer cells are characterized by abnormal DNA methylation patterns compared to normal cells[2,3]. Specifically, there is often global hypomethylation as well as gene-specific changes in promoter methylation. Some normally hypomethylated and transcriptionally active genes become hypermethylated and transcriptionally silent. Conversely, genes that are normally hypermethylated may become hypomethylated and transcriptionally active. Unfortunately, the specific mechanism by which cancer cells acquire and maintain aberrant methylation is still unknown. Three DNA methyltransferase (DNMT) enzymes found in eukaryotic cells catalyze cytosine methylation: DNMT1, DNMT3A, and DNMT3B. DNMT1 is generally regarded as the main cellular maintenance methylase, whereas DNMT3A and DNMT3B are *de novo* methylases able to establish new methylation patterns at sites that have not been previously methylated[4–6]. Interestingly, many aberrant transcripts of *DNMT3B* are highly expressed in cancer cells[7,8]. One of the most common of these transcripts, *DNMT3B7*, contains 94 bp of intron 10 sequence leading to an early stop codon and the formation of a truncated protein. Ostler and colleagues demonstrated that *DNMT3B7* is expressed in many different cancer cell types, including breast cancer[7]. Furthermore, expression of *DNMT3B7* correlates with global changes in gene expression due to altered promoter methylation. Specifically, expression of *DNMT3B7* in 293 cells led to hypermethylation of the promoter region of *CDH1/E-cadherin*[7]. Loss of *CDH1* expression is a hallmark of tumor progression to a more aggressive state and may provide a mechanism by which DNMT3B7 functions in cancer cells[9–11]. Additional studies have described changes in cellular phenotypes as a result of aberrant expression of *DNMT3B7*[7,8,12]. Because of the clinical importance of breast cancer to women’s health, we studied the mechanism(s) by which DNMT3B7 alters cell phenotype specifically within breast tumor cells.

## Materials and Methods

### TCGA analysis

Breast invasive carcinoma (BRCA) RNAseqV2 and clinical data were obtained from The Cancer Genome Atlas (TCGA) data portal. Data were organized and processed using a custom Python script and Microsoft Excel (Redmond, Washington) to analyze expression of the retained 94bp sequence of intron 10 that is specific to *DNMT3B7*. Analyses were conducted on all available patient data. Clinical staging was measured as stage I (combination of stage I, stage IA, and stage IB), stage II (combination of stage II, stage IIA, and stage IIB), stage III (combination of stage IIIA, stage IIIB, and stage IIIC), or stage IV. Each molecular subtype was defined as follows from immunohistochemical analysis found in the BRCA clinical database: luminal A (ER positive and/or PR positive and HER2 negative), luminal B (ER positive and/or PR positive and HER2 positive), triple negative/basal like (ER negative, PR negative, and HER2 negative), and HER2 type (ER negative, PR negative, and HER2 positive).

### Cells and culture conditions

MCF-7 and T-47D breast cancer cell lines were obtained from ATCC. All cells were cultured at 37°C and 5% CO_2_ in complete media containing RPMI 1640 (Life Technologies, Grand Island, NY), 10% FBS (Life Technologies) and 1% Penicillin-Streptomycin (Sigma-Aldrich, St. Louis, MO). Cells were stably transfected with 1 μg of HA-tagged DNMT3B7[7] or a vector-only control (Life Technologies) using Effectene per manufacturer protocols (Qiagen, Valencia, CA). Transfected cells were selected with G418 (Life Technologies) for 3 weeks. DNMT3B7 expression was verified by immunoblot analysis and only cell lines that showed expression levels comparable to those seen in MDA-MB-231 cells were utilized for further experimentation. All cells were harvested at ~80% confluence.

### Electrophoresis and immunoblotting

Whole cell lysate collection and immunoblotting were performed as previously described[13]. Cytoplasmic and nuclear extracts were collected as previously described[7]. All lysates contained protease inhibitor (Sigma-Aldrich) and phosphatase inhibitor (PhoSTOP, Roche, Indianapolis, IN). Blots were incubated with E-cadherin (1:1000, Cell Signaling, Danvers, MA), DNMT3B T-16 (1:250, Santa Cruz Biotechnology, Dallas, TX), topoisomerase (1:1000, Abcam, Cambridge, MA), or actin (1:5000, Millipore, Temecula, CA) and incubated in the appropriate secondary antibody (1:2000, Bio-Rad, Hercules, CA). Protein was visualized using chemiluminescent spray (Denville, South Plainfield, NJ) and imaged using a FOTO/Analyst Luminary FX system (Fotodyne, Hartland, WI).

### Sodium bisulfite treatment and methylation-specific PCR

Genomic DNA was isolated (Promega, Madison, WI) and bisulfite treated according to manufacturer protocols (CpGenome, Millipore). Methylation-specific primers to the promoter region of *E-cadherin* were developed using the MethPrimer freeware[14], forward primer: 5-GAATTGTAAAGTATTTGTGAGTTTG-3’ and reverse primer: 5’-AATACCTACAACAACAACAACAAC-3’. The 168 bp PCR product contained 15 CpGs and included the start AUG. PCR products were purified (Promega) and sequenced (ACGT, Inc., Wheeling, IL). Relative size of methylated C:T peaks was measured to determine methylation at each CpG.

### Immunofluorescence

Cells were plated on glass coverslips and immunofluorescence was performed as previously described[15] using a 1:50 dilution of β-catenin antibody (pSer^675^, Cell Signaling) and 1:500 dilution of Alexa Fluor 488 (Life Technologies). Coverslips were mounted on glass slides using ProLong Gold Antifade Reagent (Life Technologies) and imaged using a Leica DM4000 microscope and DFC360 FX Camera with AF6000 software (Leica Microsystems, Buffalo Grove, IL).

### *In vitro* cell adhesion assay

To measure cell adhesion, 5×10^4^ cells were plated in a 6-well dish and allowed to adhere for 1 hour. Cells were subsequently washed with PBS and counted to determine adhesive ability.

### *In vitro* cell proliferation assay

Cells were plated in a 6-well dish at a density of 1×10^6^ cells/well. All cells were counted and re-plated at the same density (1×10^6^ cells/well) twice a week for 3 weeks in order to avoid over-growth on the dish. The total number of cells was calculated by adding each subsequent count to the previous measurement.

### Soft agar assays

Soft agar assays were performed as previously described to measure anchorage-independent growth[16].

### Statistical analysis

Pairwise statistical significance was determined by a Student’s T-Test while comparisons among groups was analyzed with a one-way ANOVA with Tukey’s multiple comparisons using SigmaStat software (Systat, Chicago, IL). For immunoblots, densitometry was performed to determine relative expression levels using Photoshop (Adobe). Each blot was repeated three times and densities of individual bands were analyzed, normalized to the corresponding loading control, and finally normalized to control cells (set at 1.0). All experiments were performed in triplicate.

## Results

### *DNMT3B7* expression is elevated in breast tumor tissues at an early clinical stage

**Figure 1:**
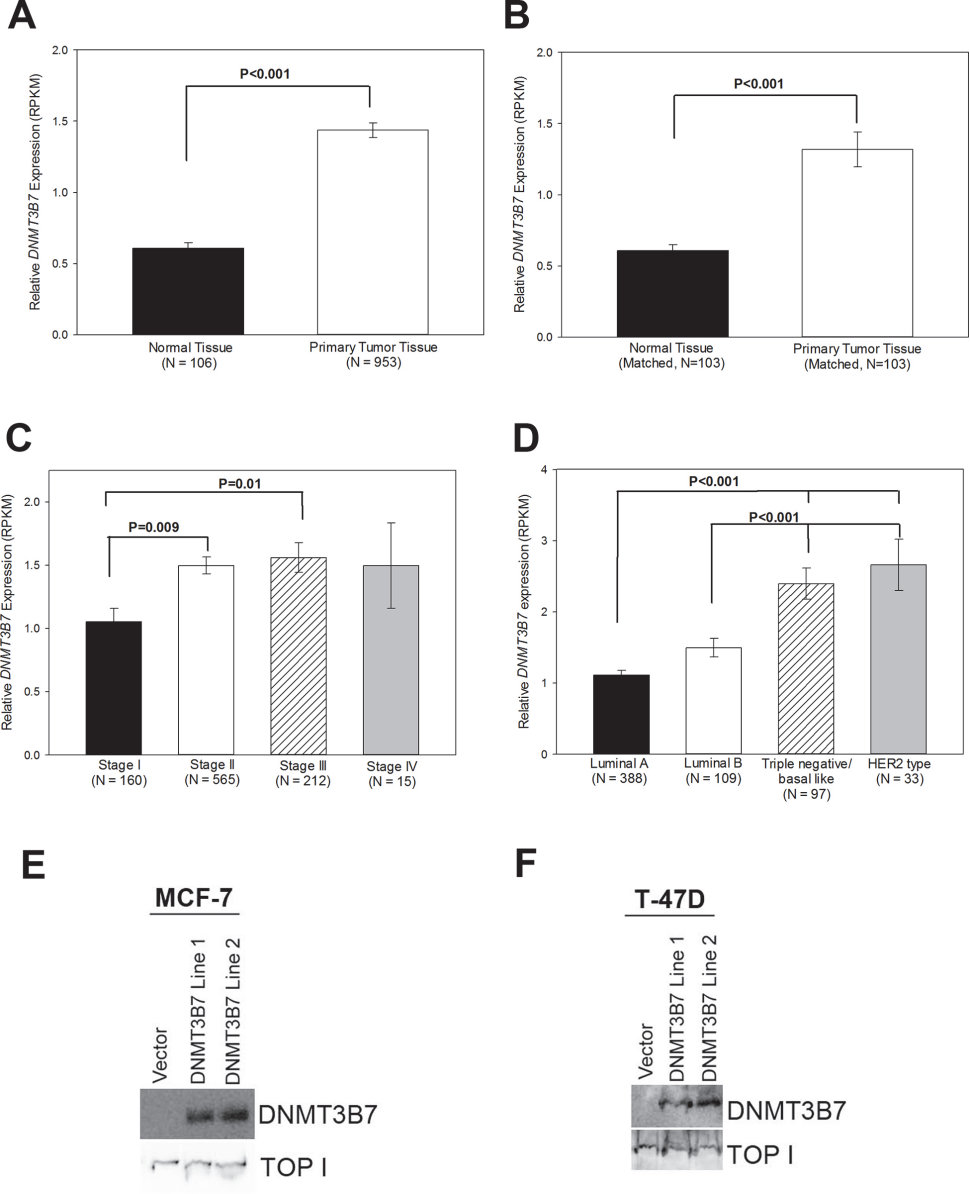
*DNMT3B7* expression in breast cancer. *DNMT3B7* reads per kilobase per million (RPKM) values of A) normal tissue versus primary tumors in unmatched patient samples or B) matched normal tissue versus primary tumor. C) *DNMT3B7* expression in all primary tumors segregated by respective tumor stages. D) *DNMT3B7* expression in all primary tumors segregated by respective molecular subtype: luminal A (ER and/or PR positive and HER2 negative), luminal B (ER and/or PR positive and HER2 positive), triple negative/basal-like (ER negative, PR negative, HER2 negative), and HER2 type (ER negative, PR negative, HER2 positive). Statistical significance is indicated on the graphs. Immunoblot analysis of nuclear lysates of (E) MCF-7 and (F) T-47D cells stably transfected with a DNMT3B7-expression construct or an empty control vector. Topoisomerase was utilized as a nuclear lysate loading control.

Previous work has demonstrated that *DNMT3B7* is highly expressed in cancer cell lines compared to normal cells[7]. To examine *DNMT3B7* expression within human tissue samples, we utilized The Cancer Genome Atlas (TCGA) and found that in over 1000 available tissue samples *DNMT3B7* expression was elevated in breast cancer samples compared to normal tissues (Fig. 1A). Furthermore, when 103 matched normal and tumor samples were analyzed, we observed the same increase in *DNMT3B7* expression, indicating that this change in expression is due to tumor progression itself and not differences within individual patient genetics (Fig. 1B). Additionally, when breast cancer patients were grouped by clinical stage, we observed that *DNMT3B7* expression increases between stage I and II, and then remains consistently high in later stages, demonstrating the aberrant transcript correlates with tumor progression to a more advanced stage (Fig. 1C). Finally, using immunohistochemical data from the TCGA, we were able to separate the breast cancer patients into one of four molecular subtypes—luminal A (ER positive and/or PR positive and HER2 negative), luminal B (ER positive and/or PR positive and HER2 positive), triple negative/basal-like (ER negative, PR negative, and HER2 negative), and HER2 type (ER negative, PR negative, and HER2 positive). It is well documented that triple negative and HER2 positive patients have a more aggressive disease phenotype that grows and spreads faster than other breast cancer subtypes[1]. Our results show an increase in *DNMT3B7* expression in triple negative and HER2 patients compared to luminal A and B groups (Fig. 1D), indicating that *DNMT3B7* expression correlates with poor prognosis in breast cancer patients.

### Expression of *DNMT3B7* in poorly invasive breast cancer cells promotes hypermethylation and down-regulation of E-cadherin

**Figure 2:**
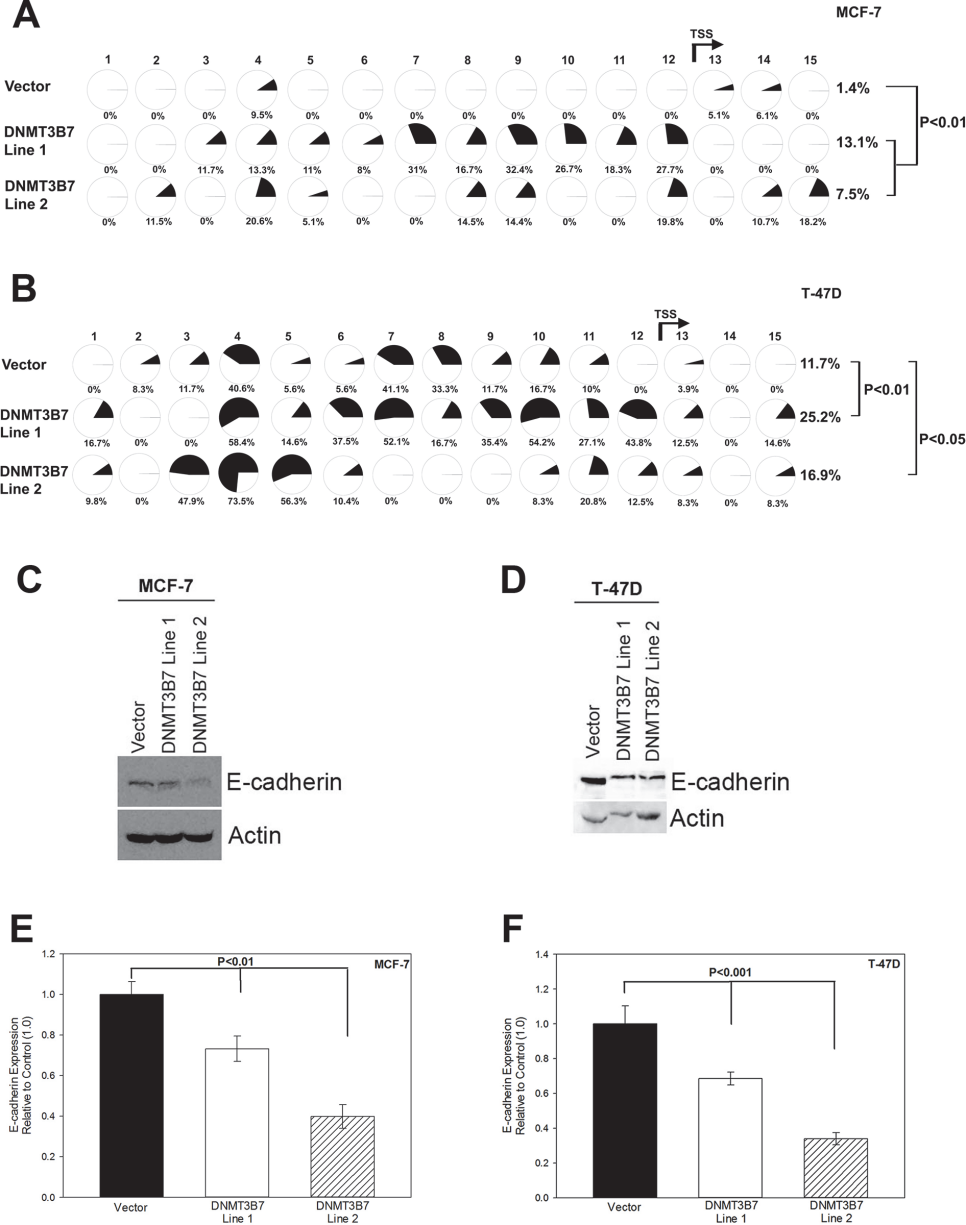
Hypermethylation and down-regulation of E-cadherin in the presence of *DNMT3B7*. Genomic DNA from stably transfected DNMT3B7-expressing cell lines and vector controls was collected, bisulfite treated, and subjected to methylation-specific PCR analysis. Primers were designed to incorporate 15 CpGs within an island encompassing the promoter region, exon 1, and the translation start site (TSS, indicated by the arrow between CpG 12 and 13). Dark shading in pie charts indicates percent of methylated C:T ratio at 15 CpG sites in (A) MCF-7 and (B) T-47D cells expressing DNMT3B7 or a control vector. Immunoblot analysis of E-cadherin expression compared to an actin loading control in (C) MCF-7 and (D) T-47D cells correlates with methylation data. Quantitative analysis using densitometry of representative blots is shown for (E) MCF-7 and (F) T-47D cells. Statistical significance is indicated on the graphs.

The clinical studies described above correlated with previous data showing increased expression of *DNMT3B7* at both the gene and protein level in the highly invasive/metastatic breast cancer cell line, MDA-MB-231, compared to the poorly invasive MCF-7 cell line, two cell lines representing the opposite extremes of breast cancer progression[7](data not shown). Although these data indicate a potential role for *DNMT3B7* in tumor progression, the exact mechanism by which it functions is unclear. To that end, we stably transfected *DNMT3B7* into two poorly invasive breast cancer cell lines, MCF-7 and T-47D, to determine the role of *DNMT3B7* in breast cancer progression (Fig. 1E-F). Previous studies have shown that expression of *DNMT3B7* in fibroblasts led to changes in cytosine modifications of *CDH1*[7], a well-known hallmark of tumor progression[9–11]. Therefore, we used bisulfite sequencing, an indirect measure of DNA methylation, to examine cytosine modifications of the *CDH1* promoter in our stably-transfected cell lines. We observed hypermethylation of a CpG island within the promoter region of *CDH1* in our MCF-7 (Fig. 2A) and T-47D (Fig. 2B) cell lines expressing *DNMT3B7*. It is important to note that there are variations in the level of hypermethylation between cell lines due to heterogeneity within the population. The CpG island included the translation start site (TSS) of *CDH1* indicating the potential for down-regulation at the protein level. Subsequent examination of E-cadherin protein expression in our cell lines via Western blot and densitometric quantification indicated a loss of E-cadherin expression in the presence of DNMT3B7 (Fig. 2C-F). Taken together, these data demonstrate that DNMT3B7 expression in breast cancer cell lines leads to hypermethylation and down-regulation of *CDH1*.

### β-catenin localization is altered in cells expressing *DNMT3B7*

**Figure 3:**
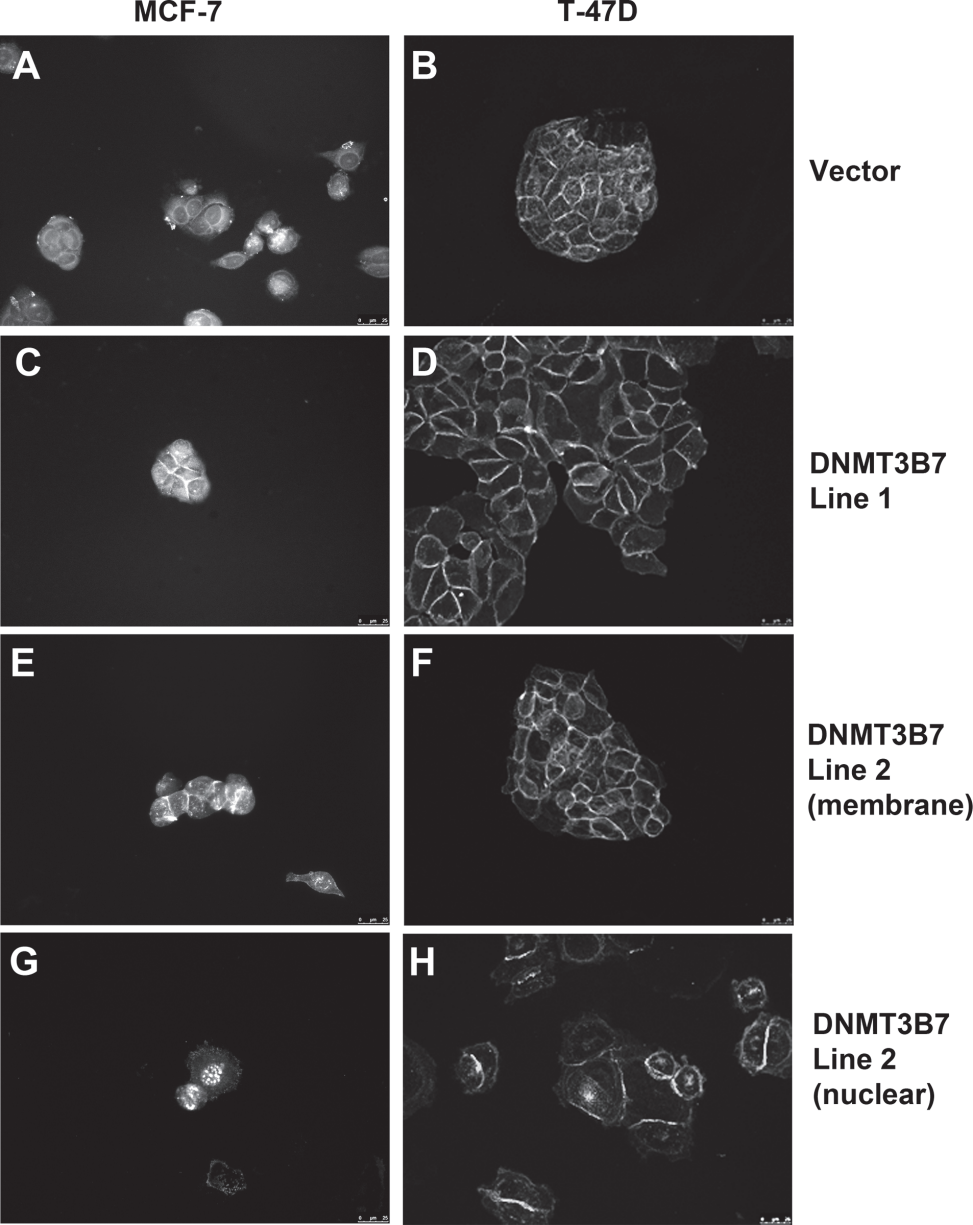
Expression of *DNMT3B7* alters β-catenin localization in breast cancer cells. Immunofluorescence of β-catenin in MCF-7 (left panel) and T-47D (right panel) cells shows membrane staining in vector controls (A-B) as well as in DNMT3B7-expressing cells (C-F). Nuclear localization of β-catenin is only observed in cells expressing DNMT3B7 (G-H).

It is well documented that a loss of E-cadherin expression can lead to altered localization of one of its binding partners, β-catenin, which correlates with poor clinical prognosis[17–22]. Under normal conditions with high levels of E-cadherin, β-catenin is expressed at the membrane to participate in cell adhesions. However, upon loss of E-cadherin expression, β-catenin translocates to the nucleus and acts as a transcription factor to promote cell proliferation. Therefore, we examined the localization of β-catenin in our cells expressing *DNMT3B7* and observed strong membrane staining in all of our cell lines (Fig. 3A-F). However, we also observed nuclear localization of β-catenin in 7% of MCF-7 and 10% of T-47D cells expressing *DNMT3B7*, which was not seen in control cells (Fig. 3G-H). This staining corresponded with the decrease in E-cadherin expression described above and may indicate a mechanism by which DNMT3B7 promotes tumor progression by changes in cell adhesion and proliferation in breast cancer cells.

### *DNMT3B7* expression promotes adhesion turnover, cell proliferation, and anchorage-independent growth in breast cancer cells

**Figure 4:**
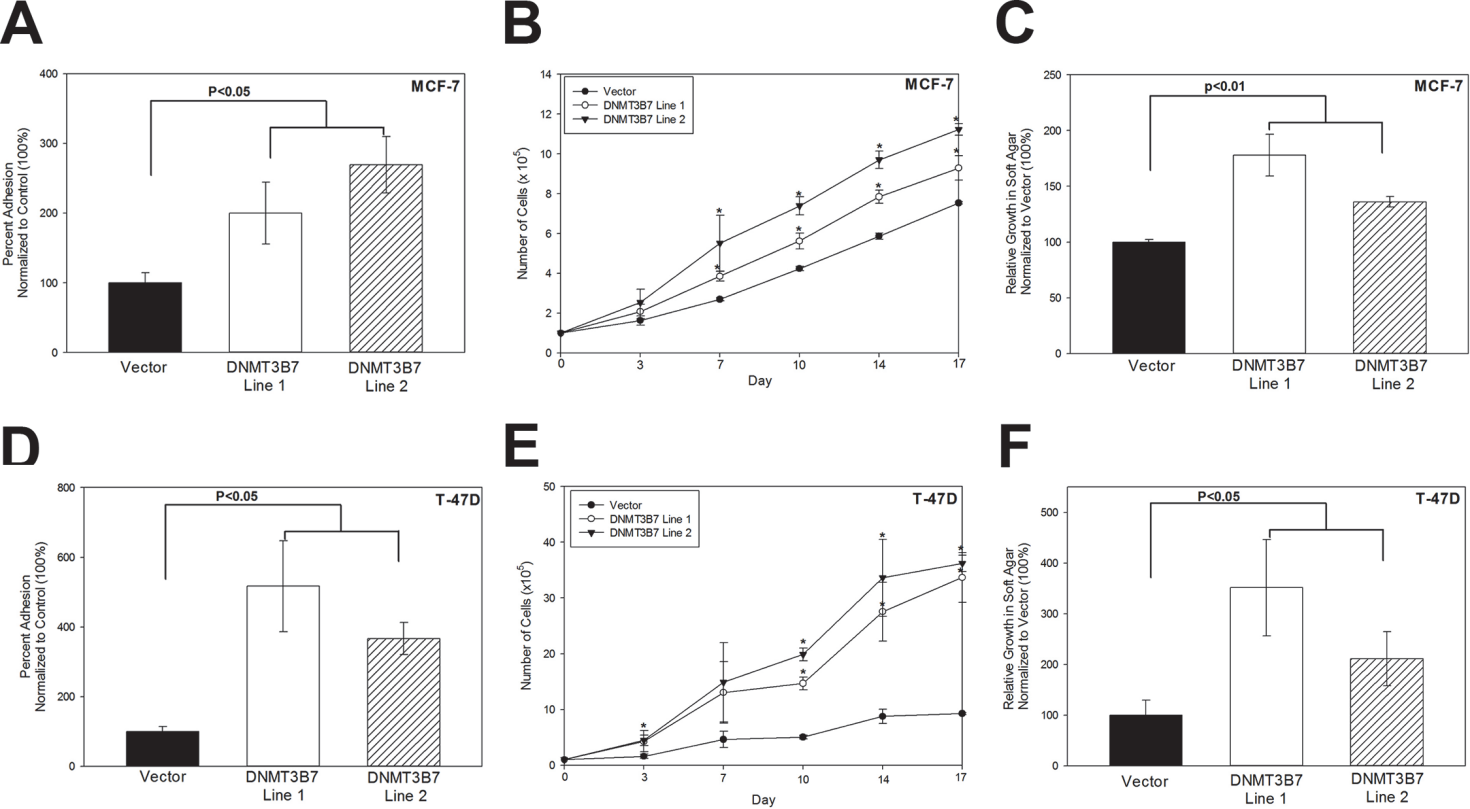
*DNMT3B7* regulates cell adhesion, proliferation, and growth in soft agar. A-C) MCF-7 or (D-F) T-47D cells stably expressing DNMT3B7 or a control vector were measured for changes in adhesion, proliferation, and growth in soft agar. A, D) Adhesion was measured as the number of cells that could adhere to a 6-well dish in 1 hour normalized to the control (100%). B, E) Cells were plated on a dish and counted twice a week for 3 weeks to measure proliferative ability. C, F) Anchorage-independent growth was determined after cells were grown in soft agar for 2 weeks, stained with crystal violet to visualize colonies, counted, and normalized to the control vector. Statistical significance is indicated on the graphs. * represents statistical significance, *p*<0.05.

The ability of cells to create and subsequently break strong cell adhesions is a key factor in progression to a more motile and aggressive phenotype. Furthermore, previous studies have demonstrated that β-catenin expression correlates with changes in cell adhesion and proliferation[23–25]. Because of the changes we observed in E-cadherin and β-catenin expression, we tested the ability of DNMT3B7 to alter cell adhesion formation and found increased adhesion turnover in cells expressing *DNMT3B7* compared to controls (Fig. 4A and 4D). Cell proliferation was also measured as an indicator of tumor progression to a more aggressive phenotype. MCF-7 (Fig. 4B) and T-47D (Fig. 4E) cells stably expressing *DNMT3B7* showed increased cell proliferation/growth over a 3-week period compared to controls indicating the progression to a more aggressive cancer phenotype. Finally, an important indicator of tumor progression is the ability of cancer cells to grow in an anchorage-independent manner. We observed increased colony growth in soft agar in MCF-7 (Fig. 4C) and T-47D (Fig. 4F) cells expressing *DNMT3B7* compared to empty vector controls. Taken together, these data demonstrate that expression of DNMT3B7 promotes a more aggressive phenotype in breast cancer cells through changes in cell adhesion, proliferation, and anchorage-independent growth.

## Discussion

**Figure 5:**
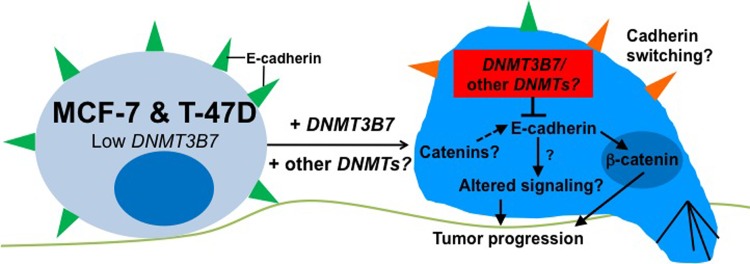
Putative model of *DNMT3B7* action in poorly invasive breast cancer cells. MCF-7 and T-47D cell lines normally have low levels of DNMT3B7 and high expression of E-cadherin. Upon stable transfection of *DNMT3B7* we observe inhibition of E-cadherin, localization of β-catenin to the nucleus, and tumor progression to a more aggressive phenotype. Future studies will examine the potential for cadherin switching in the presence of DNMT3B7 as well as changes in E-cadherin binding partners, such as catenins, and downstream signaling pathways which may promote tumor progression. Studies examining the role of other aberrant *DNMT*s will also be imperative to fully understand the role of aberrant *DNMT* transcripts in breast cancer progression.

It is imperative that we understand the cellular and molecular mechanisms by which tumors develop in order to aid in the development of novel therapeutics. To that end, the data presented here demonstrate that expression of a truncated DNA methyltransferase, *DNMT3B7*, is increased in breast cancer patient tissues compared to normal tissues and correlates to a worse clinical prognosis. In addition, we determined a molecular mechanism by which DNMT3B7 promotes tumor progression in breast cancer cells through hypermethylation and loss of *CDH1/E-cadherin* expression, altered β-catenin localization, and subsequent changes in cell adhesion, proliferation, and growth in soft agar (Fig. 5).

Interestingly, while most studies have demonstrated that DNMT3B7 promotes tumor progression in cancer cells[7,12], it has been shown to have the opposite effect in neuroblastoma cells[8]. Indeed, Ostler and colleagues showed that expression of *DNMT3B7* in neuroblastoma led to decreased proliferation and tumor progression compared to controls. The E-cadherin results shown here lead to an interesting proposition that may explain the differing results seen in our work and this previous study. It is well documented that loss of E-cadherin expression is a hallmark of breast cancer progression and a potential sign of an epithelial-to-mesenchymal transition (EMT)[26]. In breast cancer cells, cadherin switching between the normal E-cadherin expression and aberrant N-cadherin is an important indicator of tumor progression and prognosis[27]. However, in neuroblastoma cells, the opposite effect, a mesenchymal-to-epithelial transition, is a sign of tumor progression to a more aggressive state, concurrent with an up-regulation of E-cadherin and down-regulation of the normal N-cadherin. In both cases, *DNMT3B7* expression is inversely correlated with E-cadherin expression and may provide a link to its mechanism of action in cancer cells. Further studies to examine cadherin switching in cells expressing *DNMT3B7* and determine a potential role for the aberrant transcript in EMT are ongoing (Fig. 5).

The results described above indicate that inhibition of E-cadherin expression corresponds to the localization of β-catenin in the nucleus and subsequent changes in cell adhesion and proliferation. However, it is possible that other signaling pathways are altered due to the loss of E-cadherin expression which may have further consequences for these cells. Although we were able to show changes in β-catenin localization here, no studies have examined the expression or function of other catenins in cells expressing *DNMT3B7*. It is also possible that signals downstream of E-cadherin have been altered which could provide additional mechanisms by which aberrant DNMTs function in breast cancer cells. While changes to an E-cadherin mediated signaling pathway have been observed in breast cancer cell lines, it is not know at this time if these changes are regulated by DNMT3B7[28]. Future studies in our laboratory will examine the effects of aberrant DNMTs on various signaling pathways within breast cancer cells (Fig. 5).

Finally, although this study has focused on the role of DNMT3B7 in breast cancer cells, there are many other aberrant *DNMT3B* transcripts that are expressed in cancer cells[8]. It is imperative that we understand the role of each of these aberrant transcripts, functioning together and separately, in order to have a better understanding of how these transcripts contribute to transformed phenotypes. Taken together, the data presented here provide a novel mechanism by which DNMT3B7 functions in breast cancer cells to promote a more aggressive phenotype and may provide direction for novel therapeutics in the future.
